# Protective Effects of Adenosine Triphosphate and Flunarizine on Erlotinib-Induced Ovarian Damage: An Experimental Study

**DOI:** 10.3390/life16040627

**Published:** 2026-04-08

**Authors:** Arzu Yavuz, Kemine Uzel, Esra Tuba Sezgin, Mehmet Kuzucu, Nesrin Yılmaz, Gülcenaz Yazici, Engin Hendem, Halis Süleyman

**Affiliations:** 1 Department of Obstetrics and Gynecology, SBU Kocaeli City Hospital, Kocaeli 41380, Turkey; drarzuyavuz@gmail.com (A.Y.); kemineuzel@hotmail.com (K.U.); 2 Anesthesia Program, Vocational School of Health Services, Erzincan Binali Yıldırım University, Erzincan 24030, Turkey; esra.demir@erzincan.edu.tr; 3 Department of Biology, Faculty of Arts and Sciences, Erzincan Binali Yıldırım University, Erzincan 24030, Turkey; mehmetkuzucu@gmail.com; 4 Department of Obstetrics and Gynecology, Erzincan Binali Yıldırım University, Erzincan 24030, Turkey; nesrin_6554@hotmail.com; 5 Department of Histology and Embryology, Faculty of Medicine, Erzincan Binali Yıldırım University, Erzincan 24030, Turkey; gnyazici@erzincan.edu.tr; 6 Department of Medical Oncology, Mengucek Gazi Education and Research Hospital, Erzincan Binali Yıldırım University, Erzincan 24030, Turkey; enginhende61@gmail.com; 7 Department of Pharmacology, Faculty of Medicine, Erzincan Binali Yıldırım University, Erzincan 24030, Turkey

**Keywords:** ATP, erlotinib, flunarizine, ovarian damage

## Abstract

(1) Background: Erlotinib is a tyrosine kinase inhibitor (TKI) widely used in cancer therapy; however, its potential adverse effects on ovarian tissue have not been fully elucidated. The present study aimed to investigate erlotinib-induced ovarian injury and to evaluate the protective effects of adenosine triphosphate (ATP) and flunarizine, administered alone or in combination, using biochemical and histopathological analyses in a rat model. (2) Methods: Thirty female rats were randomly allocated into five groups (n = 6 per group): healthy control, erlotinib, ATP + erlotinib, flunarizine + erlotinib, and ATP + flunarizine + erlotinib. ATP (5 mg/kg, intraperitoneal) and flunarizine (5 mg/kg, oral gavage) were administered daily for two weeks, while erlotinib (5 mg/kg) was given orally every two days for two weeks. Ovarian tissues were collected for oxidative stress analysis and histopathological evaluation, and blood samples were obtained for the measurement of serum prolactin and AMH levels. (3) Results: Erlotinib administration resulted in significant oxidative stress and histopathological alterations in ovarian tissue, accompanied by a reduction in serum AMH levels, while prolactin levels remained unchanged. Treatment with ATP or flunarizine partially attenuated these alterations. (4) Conclusions: Combined ATP and flunarizine administration showed stronger protective effects, improving biochemical parameters and preserving ovarian histology, suggesting a protective role against erlotinib-induced ovarian injury.

## 1. Introduction

Erlotinib is a tyrosine kinase receptor inhibitor approved for the treatment of non-small cell lung cancer and advanced pancreatic cancer. By inhibiting the epidermal growth factor receptor (EGFR), it interferes with EGFR-mediated signaling pathways that play key roles in cellular differentiation, proliferation, and angiogenesis [1]. In addition to its use in non-small cell lung cancer and advanced pancreatic cancer, erlotinib is also used as a second-line oral antineoplastic agent in the treatment of esophageal and ovarian carcinomas [2]. However, adverse effects such as blepharitis, dry eye, diarrhea, nephrotoxicity, hepatotoxicity, and ocular toxicity have been reported during treatment [2,3]. Although targeted anticancer agents are generally considered to cause fewer side effects than conventional chemotherapeutics, suppression of multiple intracellular mechanisms may still induce ovarian toxicity [4]. EGFR activity in the ovary is required for oocyte maturation, cumulus expansion, and ovulation [5]. Most tyrosine kinase and receptor inhibitors, including erlotinib, may affect both ovarian and testicular functions, particularly processes such as spermatogenesis, oogenesis, primordial follicle activation, folliculogenesis, and corpus luteum formation and maturation [6]. The literature indicates that reactive oxygen species (ROS) are among the key factors in the pathogenesis of ovarian damage. The adverse effects of erlotinib and other chemotherapeutic agents have been associated with oxidative stress and excessive mitochondrial ROS production [7]. Cytoplasmic calcium (Ca^2+)^ concentration, mitochondrial membrane depolarization, and ROS levels have been reported to increase with erlotinib administration [8]. Moreover, increased intracellular Ca^2+^ content may lead to reduced ATP levels [9]. Decreased ATP synthesis may increase ROS production, alter mitochondrial membrane permeability, and disrupt Ca^2+^ homeostasis [10]. Although there are indications regarding the roles of ROS and Ca^2+^ in erlotinib-associated ovarian damage, the available evidence remains limited.

Flunarizine, evaluated in this study for its potential protective effects against possible erlotinib-induced ovarian damage, is a prophylactic treatment option for episodic migraine. It is a Ca^2+^ antagonist that inhibits slow Ca^2+^ channels [11]. As a piperazine derivative, flunarizine also blocks sodium (Na^+^) channels [12]. Flunarizine has been reported to prevent acute renal dysfunction and mitochondrial damage by significantly reducing total calcium while increasing antioxidant capacity and ATP levels [13]. Because mitochondrial function reflects oxygen consumption, Ca^2+^ utilization, ATP production, and regulation of the respiratory chain, these findings suggest that ATP and flunarizine may be beneficial against possible erlotinib-induced ovarian damage. To the best of our knowledge, no studies have investigated the protective effects of ATP and flunarizine against possible erlotinib-induced ovarian damage. Although erlotinib has been associated with ovarian toxicity through oxidative stress and Ca^2+^ dysregulation, the underlying mechanisms, particularly the roles of mitochondrial dysfunction and ATP depletion, remain unclear. Moreover, no studies have evaluated therapeutic strategies targeting these pathways in erlotinib-induced ovarian damage. Therefore, ATP and flunarizine, due to their roles in cellular energy regulation and Ca^2+^ homeostasis, may represent promising protective agents. To the best of our knowledge, this is the first study to investigate their individual and combined effects against erlotinib-induced ovarian injury. Therefore, the aim of this study was to investigate and compare the protective effects of ATP, flunarizine, and their combination against possible erlotinib-induced ovarian damage in rats.

## 2. Materials and Methods

### 2.1. Animals

The study was conducted on female albino Wistar rats. A total of 30 rats weighing 266–279 g were used. All animals were obtained from the Experimental Animals Application and Research Center of Erzincan Binali Yıldırım University. Before the experiment, animals were housed in groups (n = 6) under standard laboratory conditions (21–22 °C, 30–70% humidity, 12 h light/12 h dark cycle) and provided with water and standard pellet feed ad libitum. Animals were randomly allocated to experimental groups in a manner aimed at minimizing selection bias. The sample size (n = 6 per group) was determined based on previously published studies with similar experimental designs. The estrous cycle of the animals was not controlled. All procedures were approved by the Erzincan Binali Yıldırım University Local Ethics Committee for Animal Experiments (meeting date: 25 December 2025 and number: 2025/12) and were performed in accordance with European Parliament Directive 2010/63/EU (Approval No. 2016-24-199) and ARRIVE guidelines [14].

### 2.2. Chemicals

Flunarizine (5 mg tablet; Johnson & Johnson Sıhhi Malzemeler San. ve Tic. Ltd. Şti., Istanbul, Türkiye), erlotinib (Tarceva^®^, 100 mg tablet; Roche, Istanbul, Türkiye), thiopental sodium (IE Ulagay, Istanbul, Türkiye), and adenosine triphosphate (ATP; Zdorove Narodu, Ukraine) were used in the experiments. All chemicals were of analytical grade and prepared freshly before use.

### 2.3. Experimental Groups

Rats were allocated into five groups: healthy control (HG), erlotinib alone (ERG), ATP + erlotinib (AERG), flunarizine + erlotinib (FERG), and ATP + flunarizine + erlotinib (AFEG) (n = 6 per group).

### 2.4. Experimental Procedure

The doses and administration routes of ATP [15] and flunarizine [16] were selected according to previous experimental studies demonstrating their protective effects in rat toxicity models. Erlotinib doses between 5 and 50 mg/kg have been used in experimental toxicity models [17]. In the present study, 5 mg/kg was selected to induce subclinical ovarian injury without causing excessive systemic toxicity, allowing evaluation of protective interventions. ATP (5 mg/kg) was administered intraperitoneally (i.p.) to the AERG group. Flunarizine (5 mg/kg) was administered orally by gavage to the FERG group. Distilled water was administered as a vehicle to the HG and ERG groups. The AFEG group received both flunarizine and ATP at the same doses and via the same routes. One hour after administration of ATP and/or flunarizine, erlotinib (5 mg/kg) was administered orally by gavage to the ERG, AERG, FERG, and AFEG groups. This interval was chosen to ensure sufficient systemic availability of the pretreatments before erlotinib administration. ATP and flunarizine were administered once daily for two weeks. Erlotinib was administered once every two days for two weeks. At the end of the experimental period, rats were sacrificed under high-dose anesthesia (thiopental sodium, 50 mg/kg). Ovarian tissues were excised for biochemical analyses of oxidant/antioxidant parameters and for histopathological examination. Biochemical and histopathological findings were compared across groups.

### 2.5. Biochemical Analyses

#### 2.5.1. Sample Preparation

After recording ovarian tissue weights, the samples were cut into small pieces, snap-frozen in liquid nitrogen, and homogenized using a mortar and pestle. The homogenates were vortexed for 10 s in phosphate-buffered saline (PBS, pH 7.4) at a ratio of 1:10 (*w*/*v*), followed by centrifugation at 10,000 rpm for 20 min. The supernatants were collected and stored at −80 °C until biochemical analysis.

#### 2.5.2. Ovarian Tissue MDA, tGSH, SOD, CAT, and Protein Analyses

Ovarian tissue MDA, tGSH, and SOD levels were measured using rat enzyme-linked immunosorbent assay (ELISA) kits according to the manufacturer’s instructions (product numbers 10009055, 703002, and 706002; Cayman Chemical Company, Ann Arbor, MI, USA). Catalase (CAT) activity was determined using the method described by Goth [18]. Tissue protein levels were measured according to the Bradford method [19].

#### 2.5.3. Serum Prolactin Measurement

Serum prolactin levels were measured using an automated immunoassay analyzer (Architect i2000SR; Abbott Laboratories, Abbott Park, IL, USA) with the Architect Prolactin kit (Ref. No. 7K76-25) according to the manufacturer’s instructions.

#### 2.5.4. Serum Anti-Müllerian Hormone Measurement

Serum AMH levels were measured using an AMH kit (UNICELL-AMH; YHLO Biotech Co., Ltd., Shenzhen, China) based on an immunochromatographic sandwich assay that generates a fluorescent signal proportional to the AMH concentration. According to the manufacturer, the analytical sensitivity and measurement range of the assay were 0.1–25.0 ng/mL.

### 2.6. Histopathological Procedure

#### 2.6.1. Histopathological Processing and Staining

Ovarian tissue samples were fixed in 10% neutral-buffered formaldehyde for 72 h and subsequently washed with tap water for 24 h. The tissues were then processed through a graded series of ethanol solutions (70%, 80%, 90%, and 100%) for dehydration, cleared in xylene, and embedded in paraffin. Sections of 4 μm thickness were cut from paraffin blocks and stained with hematoxylin and eosin (H&E) [20]. The stained sections were examined and digitally photographed using a light microscope equipped with DP2-SAL software (Olympus Inc., Tokyo, Japan).

#### 2.6.2. Histopathological Scoring

For semiquantitative evaluation, one central region and five peripheral cortical regions were systematically selected from serial sections for each specimen to minimize sampling bias, and degenerative changes were scored within these areas. Ovarian tissue damage was assessed based on follicular degeneration, interstitial edema, vascular dilatation/congestion, and inflammatory cell infiltration. Each parameter was scored using a predefined four-point scale: 0, no damage; 1, mild damage; 2, moderate damage; and 3, severe damage. Representative histological reference features corresponding to each score were used to ensure consistency across evaluations. From serially sectioned samples taken from each rat, six non-overlapping fields were selected, and primordial follicles, developing follicles (primary, secondary, and tertiary follicles were classified as developing follicles), atretic follicles, and corpus luteum were counted at 100 × magnification. To avoid double-counting, follicles were recorded only when the oocyte nucleus was clearly visible within the section. Histopathological assessment and scoring were performed by an experienced histologist who was blinded to the experimental groups. All evaluations were conducted using standardized criteria applied uniformly to all specimens.

### 2.7. Statistical Analyses

All statistical analyses were performed using SPSS software (version 18.0; SPSS Inc., Chicago, IL, USA). Data were expressed as mean ± standard deviation (SD). Differences among groups were evaluated using one-way analysis of variance (ANOVA), followed by Fisher’s least significant difference (LSD) post hoc test for multiple comparisons. Histopathological data were analyzed using the Kruskal–Wallis test, followed by the Mann–Whitney U test for pairwise comparisons. The assumptions of normality and homogeneity of variances were assessed prior to ANOVA. Exact *p* values are reported where appropriate. A *p* value < 0.05 was considered statistically significant.

## 3. Results

### 3.1. Biochemical Findings

#### 3.1.1. MDA and tGSH Levels in Ovarian Tissue

**Figure 1:**
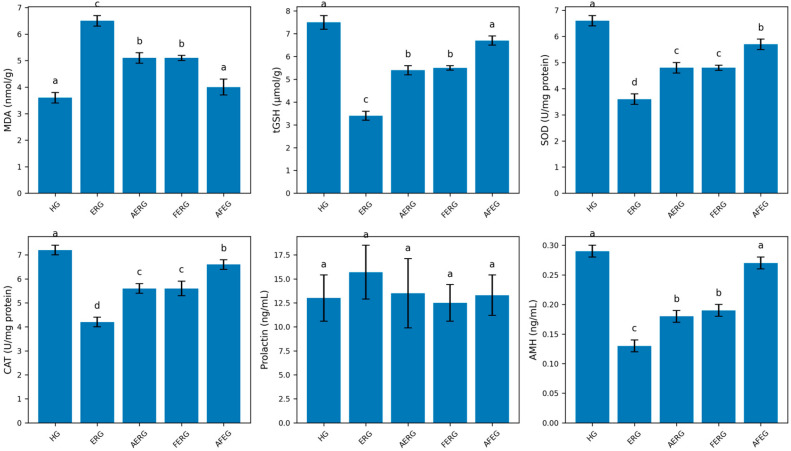
Effects of ATP and flunarizine on erlotinib-induced oxidative stress in ovarian tissue. Levels of malondialdehyde (MDA), total glutathione (tGSH), superoxide dismutase (SOD), catalase (CAT), prolactin, and anti-Müllerian hormone (AMH) were measured across experimental groups. Data are presented as mean ± SD (n = 6). Different letters above the bars indicate statistically significant differences between groups (*p* < 0.05). HG: healthy group; ERG: erlotinib group; AERG: ATP + erlotinib group; FERG: flunarizine + erlotinib group; AFEG: ATP + flunarizine + erlotinib group.

As shown in Figure 1, erlotinib markedly increased ovarian tissue MDA levels. The difference in MDA levels between the healthy and erlotinib groups was highly significant (*p* < 0.0001). ATP and flunarizine significantly suppressed the erlotinib-induced increase in MDA levels (*p* < 0.01). However, the difference in MDA levels between the ATP and flunarizine groups was not statistically significant (*p* > 0.05). The AFEG group most effectively prevented the erlotinib-induced increase in MDA. In the AFEG group, MDA levels were close to those of the HG, and the difference was not statistically significant (*p* > 0.05). The AFEG treatment was more effective than ATP or flunarizine alone in preventing the increase in MDA levels (*p* < 0.01).

In addition, ovarian tGSH levels in erlotinib-treated animals were significantly lower than those in the *HG* (*p* < 0.0001). ATP and flunarizine significantly prevented the erlotinib-induced reduction in tGSH levels (*p* < 0.01). The difference in tGSH levels between the AERG and FERG groups was not statistically significant (*p* > 0.05). The AFEG combination most effectively prevented the erlotinib-induced decrease in tGSH levels (*p* < 0.0001) (Figure 1).

#### 3.1.2. SOD and CAT Activities in Ovarian Tissue

As shown in Figure 1, erlotinib caused a significant decrease in SOD and CAT activities in ovarian tissue. Compared with the HG, SOD and CAT activities in the ERG were significantly lower (*p* < 0.0001). ATP and flunarizine similarly prevented the erlotinib-induced decreases in SOD and CAT activities (*p* < 0.01). AFEG more strongly suppressed the erlotinib-induced decreases in SOD and CAT activities (*p* < 0.0001). Moreover, AFEG was significantly more effective than AERG or FERG alone (*p* < 0.01).

#### 3.1.3. Serum Prolactin and Anti-Müllerian Hormone Levels

As shown in Figure 1, no statistically significant differences were observed in serum prolactin levels between the HG and any of the treatment groups (*p* > 0.05). Serum AMH levels were significantly lower in the ERG compared to the HG (*p* < 0.0001). This decrease was partially attenuated by ATP (*p* < 0.05). Although flunarizine appeared to attenuate the erlotinib-induced decrease in AMH to a slightly greater extent than ATP, the difference between the AERG and FERG groups was not statistically significant (*p* > 0.05). The AFEG group showed the greatest attenuation of the erlotinib-induced decrease in AMH (*p* < 0.0001). AMH levels in the AFEG group were significantly higher than those in the AERG and FERG groups (*p* < 0.01).

### 3.2. Histopathological Findings

**Figure 2:**
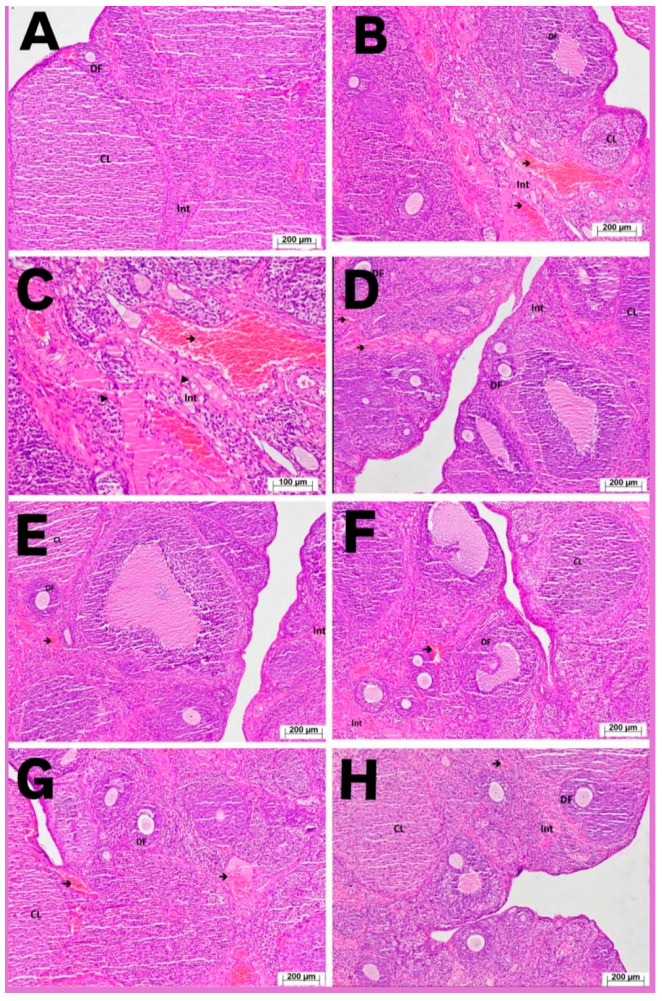
Representative H&E-stained ovarian tissue sections from the experimental groups. (**A**) Healthy group (HG) showing normal cortical and medullary structures with developing follicles (DF), corpus luteum (CL), and normal interstitial tissue (Int). (**B**,**C**) Erlotinib group (ERG) demonstrating severe vascular congestion and dilatation (arrows), marked reduction in developing follicles, interstitial edema, and inflammatory cell infiltration (arrowheads). (**D**,**E**) AERG group showing partial histological improvement with increased developing follicles, but persistent moderate interstitial edema and vascular congestion. (**F**,**G**) FERG group showing similar moderate histopathological changes, including interstitial edema and vascular congestion with an increased number of developing follicles compared with ERG. (**H**) AFEG group demonstrating near-normal ovarian architecture with preserved follicular structures and only mild vascular congestion. DF: developing follicle; CL: corpus luteum; Int: interstitial area. Arrows indicate congested blood vessels, and arrowheads indicate inflammatory cell infiltration. Scale bars: 200 μm (C: 100 μm).

Histological examination of ovarian tissues from the HG group revealed normal cortical and medullary structures. Interstitial connective tissue, corpora lutea, developing follicles, and blood vessels exhibited normal histological appearance (Figure 2A). In the ERG group, the severity of ovarian damage was graded as 3 (Table 1). Severe vascular congestion and dilatation were observed, and the number of developing follicles was markedly reduced compared with the healthy group (Figure 2B). At higher magnification, inflammatory cell infiltration was evident, and prominent edematous areas were detected in the interstitial connective tissue (Figure 2C). In the AERG group, ovarian damage was graded as 2 (Table 1). Compared with the ERG group, a higher number of developing follicles was observed. Interstitial edema persisted, and vascular dilatation and congestion were present (Figure 2D,E). In the FERG group, histopathological damage was graded as 2 (Table 1). The number of developing follicles was increased compared with the ERG but remained lower than in the HG. Interstitial edema and vascular dilatation/congestion were also observed (Figure 2F,G). In the AFEG group, cortical and medullary structures appeared histologically normal. The number of developing follicles was close to that of the healthy group (grade 0–1), interstitial connective tissue showed a normal appearance, and blood vessels exhibited normal histology. Mild congestion (grade 1) persisted (Figure 2H, Table 1).

**Figure 3:**
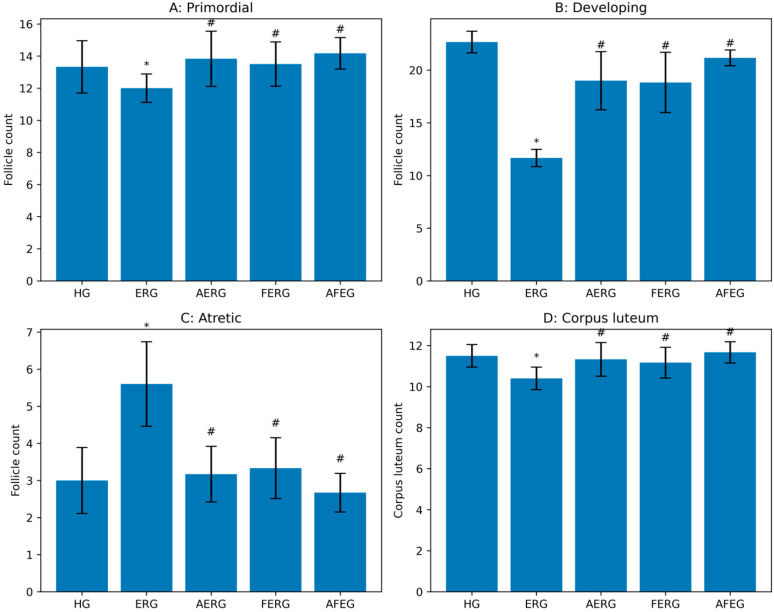
Effects of experimental treatments on ovarian follicle dynamics and corpus luteum count across groups. (**A**) Primordial follicles, (**B**) developing follicles, (**C**) atretic follicles, and (**D**) corpus luteum counts. Data are presented as mean ± SD (n = 6 per group). * *p* < 0.05 vs. HG; # *p* < 0.05 vs ERG. HG: healthy group; ERG: erlotinib group; AERG: ATP + erlotinib group; FERG: flunarizine + erlotinib group; AFEG: ATP + flunarizine + erlotinib group.

Quantitative follicle count analysis demonstrated that the number of developing follicles was significantly reduced in the ERG group compared with the HG. In contrast, atretic follicle numbers were significantly increased, indicating enhanced follicular degeneration following erlotinib exposure. Flunarizine treatment improved follicular preservation. The number of developing follicles was significantly higher in the AERG, FERG, and AFEG groups compared with the ERG group, whereas atretic follicle counts were reduced. Among the treatment groups, the AFEG group exhibited follicle counts most closely resembling those of the HG. Although primordial follicle and corpus luteum numbers showed partial improvement in treatment groups, these differences did not reach statistical significance (Figure 3).

## 4. Discussion

In this study, the protective effects of ATP, flunarizine, and their combination against erlotinib-induced oxidative ovarian damage in female rats were investigated using biochemical and histopathological methods. In addition, serum prolactin and AMH levels, which are commonly used in the evaluation of infertility, were measured. Because most patients receive combination chemotherapy, adverse outcomes such as reproductive dysfunction and infertility are frequently reported. However, only a limited number of studies have examined the mechanisms of gonadotoxicity induced by single chemotherapeutic agents, and oxidative stress has been proposed to contribute to loss of reproductive function [7].

Our findings demonstrated that ovarian MDA levels, a product of ROS-related lipid peroxidation, were significantly increased in the ERG. MDA is widely used as a marker of oxidative stress, and increased levels reflect elevated ROS [21]. In the literature, erlotinib-induced oxidative stress has been associated with increased cytoplasmic Ca^2+^, mitochondrial membrane depolarization, and enhanced ROS production [8]. Mitochondrial dysfunction is closely linked to increased ROS generation and depletion of antioxidant capacity [22]. Consistent with these reports, we observed significant reductions in non-enzymatic (tGSH) and enzymatic (SOD, CAT) antioxidants, paralleled by increased MDA in ovarian tissue following erlotinib administration. Decreases in tGSH, SOD, and CAT have been interpreted as indicating insufficient antioxidant defense [23]. These enzymatic and non-enzymatic antioxidants constitute major cellular defense mechanisms against oxidative injury [24]. Our findings indicate that, in the ERG, the oxidant–antioxidant balance shifted toward oxidants in ovarian tissue. To evaluate the potential effects of erlotinib on reproductive function, serum prolactin and AMH levels were measured. Prolactin, an anterior pituitary hormone, is known to suppress gonadotropin-releasing hormone and follicular maturation when elevated [25,26]. However, no statistically significant differences were observed in prolactin levels between the groups. Therefore, prolactin does not appear to be affected under the conditions of this study. Serum AMH levels were assessed as a marker of ovarian function. AMH is a glycoprotein secreted by granulosa cells of small follicles and is widely used as a clinical indicator of ovarian reserve [27]. We observed a significant decrease in serum AMH levels in erlotinib-treated animals. Although there are no direct data regarding the effect of erlotinib on AMH, previous studies have reported that certain tyrosine kinase inhibitors may reduce AMH levels [28]. The observed decrease in AMH levels may suggest a potential impact on ovarian reserve and reproductive outcomes [29,30,31]; however, these findings should be interpreted cautiously within the limitations of the study. ATP, which we tested against possible erlotinib-induced reproductive toxicity, is a nucleoside triphosphate composed of adenine, ribose, and three phosphate groups [32]. ATP significantly attenuated the erlotinib-induced increase in oxidants and decrease in antioxidants. As noted above, erlotinib increases intracellular Ca^2+^ levels [8], and increased intracellular Ca^2+^ may reduce ATP stores [9]. Decreased ATP synthesis can promote ROS production [10]. These mechanisms may explain why exogenous ATP administration suppressed the erlotinib-associated increase in MDA and clarified its antioxidant effect. ATP depletion is known to result in impaired mitochondrial membrane potential, increased ROS, and cellular injury [21]. No prior studies were identified that specifically investigated the effects of ATP against erlotinib-induced ovarian damage. However, ATP has been reported to reduce 5-fluorouracil-induced oxidative stress in rat ovaries via antioxidant mechanisms [33]. Although ATP significantly altered oxidant–antioxidant parameters, it did not significantly affect prolactin levels. Conversely, ATP attenuated the erlotinib-related decline in AMH. These findings indicate that prolactin levels were not associated with the observed oxidative changes under the conditions of this study. In contrast, changes in AMH levels were observed alongside alterations in oxidative status. However, these findings should be interpreted cautiously.

Flunarizine was the second agent tested for protection against possible erlotinib-induced ovarian damage. Demonstration of increased intracellular Ca^2+^ in the pathogenesis of erlotinib-induced tissue injury [8] suggested that flunarizine might be effective against erlotinib toxicity. Flunarizine blocks not only Ca^2+^ channels but also Na^+^ channels [11,12]. In our study, flunarizine significantly inhibited erlotinib-induced increases in oxidants and decreases in antioxidants in ovarian tissue. The protective effect of flunarizine against erlotinib-associated oxidative damage in ovarian tissue is considered to be related to inhibition of Ca^2+^ channels. The relationship between intracellular Ca^2+^ and ATP [9] further supports the antioxidant effect of flunarizine. Our findings are also consistent with reports that flunarizine prevents mitochondrial damage by increasing antioxidant capacity and ATP levels [13]. At the dose producing antioxidant effects, flunarizine did not alter prolactin levels, while it attenuated the erlotinib-induced decline in AMH. Previous findings have reported that hyperprolactinemia may not be associated with oxidative stress or infertility [34]. In contrast, some studies have suggested a possible relationship between AMH levels and oxidative stress [35]. In the AFEG group, which exhibited a stronger antioxidant effect, higher AMH levels were observed compared with those in the other treatment groups. However, these findings should be interpreted cautiously.

Biochemical findings related to oxidants, antioxidants, prolactin, and AMH were found to be consistent with the histopathological results and quantitative follicle count analysis. Follicular and vascular changes observed in the ovaries of erlotinib-treated animals were compatible with oxidative stress–related tissue injury, which was further supported by the marked reduction in primordial and developing follicle numbers and the increase in atretic follicles. The adverse effects of erlotinib and other chemotherapeutic agents have been reported to be associated with oxidative stress and excessive mitochondrial ROS production [7]. Oxidative stress has been defined in the literature as one of the fundamental mechanisms underlying ovarian damage [33]. ATP and flunarizine reduced erlotinib-induced ovarian injury. However, AFEG exhibited a more pronounced protective effect in terms of preservation of follicular reserve and reduction in follicular atresia. Although no previous studies have reported the protective effects of flunarizine against ovarian injury, ATP has been shown histopathologically to reduce the severity of drug-induced ovarian damage [36]. The present quantitative follicle count findings support an important role of flunarizine in preserving ovarian reserve and structural integrity following oxidative injury.

Whether erlotinib directly leads to infertility remains unclear; therefore, the present study has several important limitations. Firstly, functional fertility outcomes, such as live birth and mating success, were not evaluated. Consequently, the findings of this study should be interpreted as being limited to biochemical, histopathological, and quantitative follicle count indicators. Another limitation of this study is that the estrous cycle of the animals was not controlled. Ovarian functions and follicular dynamics are highly influenced by hormonal changes occurring throughout this cycle, which may have affected the observed results. Although changes in follicle numbers, corpus luteum structures, and oxidative stress markers provide important evidence regarding ovarian reserve and tissue architecture, these parameters do not fully reflect long-term reproductive capacity. The relatively short follow-up period further limits the assessment of whether the observed protective effects translate into sustained fertility preservation. Therefore, the findings of this study should be interpreted as reflecting short-term biochemical and histological effects in healthy rats rather than direct evidence of fertility preservation. The experimental design did not include a recognized positive control group with established antioxidant or protective efficacy, nor did it include groups receiving ATP or flunarizine alone. The absence of such comparators may limit the ability to distinguish their independent effects and to contextualize the magnitude and clinical relevance of the observed protective effects. The study was conducted in healthy, non–cancer-bearing rats, which may not fully replicate the complex systemic, metabolic, and inflammatory conditions present in cancer patients receiving erlotinib therapy. The absence of a clinically relevant cancer model may limit the translational relevance of the findings and the accurate simulation of real-world clinical scenarios. Moreover, as this study used an experimental animal model, caution should be exercised when extrapolating the results to human clinical settings. Biological differences between rats and humans may limit clinical extrapolation of the findings. In addition, the study did not specifically investigate detailed molecular mechanisms such as apoptosis, mitochondrial dysfunction, or signaling pathways. Instead, it relied solely on oxidative stress markers, which may be considered a limitation. Finally, while the present study focused on the protective effects of flunarizine against erlotinib-induced ovarian injury, different dosing regimens and alternative treatment strategies were not evaluated. Further studies are needed to better clarify the reproductive safety of erlotinib and the protective potential of flunarizine.

## 5. Conclusions

In conclusion, erlotinib administration induced oxidative stress in ovarian tissue, as demonstrated by biochemical and histopathological analyses. Erlotinib did not significantly alter serum prolactin levels, but it reduced serum AMH levels. The combined ATP and flunarizine treatment was more effective than ATP or flunarizine alone in preventing erlotinib-associated ovarian damage. These findings suggest that combined ATP and flunarizine treatment may be more beneficial in the management of erlotinib-related ovarian injury. However, our findings should be interpreted with caution, as the study relied primarily on oxidative stress markers and did not investigate detailed molecular mechanisms such as apoptosis, mitochondrial function, or signaling pathways. Therefore, further studies are needed to better elucidate the underlying mechanisms and confirm the translational relevance of these results.

## Figures and Tables

**Table 1:** Semiquantitative Histopathological Evaluation of Ovarian Tissue Damage.

Groups	Vascular Dilatation/Congestion	Interstitial Edema	Inflammatory Cell Infiltration	Follicular Loss	Total Damage Score
HG	0.0 ± 0.0 ^d^	0.0 ± 0.0 ^d^	0.0 ± 0.0 ^d^	0.0 ± 0.0 ^d^	0.0 ± 0.0 ^d^
ERG	3.0 ± 0.0 ^a^	3.0 ± 0.0 ^a^	3.0 ± 0.0 ^a^	3.0 ± 0.0 ^a^	12.0 ± 0.0 ^a^
AERG	2.0 ± 0.4 ^b^	2.0 ± 0.5 ^b^	2.0 ± 0.5 ^b^	2.0 ± 0.4 ^b^	8.0 ± 1.2 ^b^
FERG	2.0 ± 0.5 ^b^	1.5 ± 0.5 ^b^	1.5 ± 0.5 ^b^	2.0 ± 0.5 ^b^	7.0 ± 1.1 ^b^
AFEG	1.0 ± 0.3 ^c^	1.0 ± 0.3 ^c^	0.5 ± 0.3 ^c^	1.0 ± 0.3 ^c^	3.5 ± 0.8 ^c^

Histopathological damage was evaluated semi-quantitatively based on vascular dilatation/congestion, interstitial edema, inflammatory cell infiltration, and follicular loss. Scores were defined as: 0 = none, 1 = mild, 2 = moderate, and 3 = severe. Data are presented as mean ± SD (n = 6). Statistical analysis was performed using the Kruskal–Wallis test followed by the Mann–Whitney U test. Different superscript letters within the same column indicate statistically significant differences between groups (*p* < 0.05).

## Data Availability

Data from the research can be obtained from the corresponding author upon reasonable request.

## Abbreviations

The following abbreviations are used in this manuscript:

AMH	Anti-Müllerian Hormone
ATP	Adenosine Triphosphate
CAT	Catalase
EGFR	Epidermal Growth Factor Receptor
MDA	Malondialdehyde
ROS	Reactive Oxygen Species
SOD	Superoxide Dismutase
tGSH	Total Glutathione
TKI	Tyrosine Kinase Inhibitor
